# Relevance of Reversible Causes of Out-of-Hospital Cardiac Arrest: The “REBECCA” Interactive Checklist

**DOI:** 10.3390/jcm15062422

**Published:** 2026-03-21

**Authors:** Martina Hermann, Arthur Stoiber, Andreas Schmid, Thomas Hamp, Angelika De Abreu Santos, Daniel Grassmann, Mario Krammel, Josef M. Lintschinger, Stefan Ulbing, Alessa Stria, Christina Hafner

**Affiliations:** 1 Medical University of Vienna, Department of Anaesthesia, Intensive Care Medicine and Pain Medicine, Clinical Division of General Anaesthesia and Intensive Care Medicine, 1090 Vienna, Austria; martina.hermann@meduniwien.ac.at (M.H.); arthur.stoiber@meduniwien.ac.at (A.S.);; 2 Ludwig Boltzmann Institute Digital Health and Patient Safety, 1090 Vienna, Austria; 3 Emergency Medical Service Vienna, 1030 Vienna, Austria; 4 PULS—Austrian Cardiac Arrest Awareness Association, 1090 Vienna, Austria

**Keywords:** cardiac arrest, cardiopulmonary resuscitation, out-of-hospital cardiac arrest, checklist, advanced cardiac life support, prehospital emergency care, emergency medical service, cognitive aid, prehospital care, emergency medicine

## Abstract

**Background/Objectives**: Adequate cardiopulmonary resuscitation (CPR), defibrillation, and treatment of reversible causes are essential for improving the survival of patients suffering from out-of-hospital cardiac arrests (OHCAs). The Advanced Life Support (ALS) algorithm includes reversible causes for cardiac arrest. This study aimed to develop an interactive mobile checklist to identify reversible causes of OHCA (REBECCA) and evaluate their usability and usefulness among emergency physicians. **Methods**: This mixed-methods study was conducted at the Emergency Medical Service Vienna, Austria. All participants were emergency physicians from the Medical University of Vienna. An interactive mobile checklist was developed using a participatory design approach involving a focus group of 10 emergency physicians. Usability and applicability were assessed using structured questionnaires. Descriptive statistics were used to summarize participant characteristics and evaluation outcomes. **Results**: Among the included participants, 70% were specialists with a median prehospital experience of 2.0 (1.0–4.3) years. Although most participants were confident about their level of professional experience with OHCA, 85% still found the checklist to be helpful. The majority of the participants preferred the digital checklist over the paper-based checklist and appreciated its integration with the point-of-care ultrasound (POCUS) application. Although the participants did not communicate a significant need for further details on most causes, a small majority favored more information on intoxication and electrolyte disorders. **Conclusions**: The majority of the included emergency physicians found the REBECCA checklist helpful regardless of training level, whereas almost no physician needed further detailed information on the reversible causes. Our findings underscore the potential importance of future investigations aiming to reduce the cognitive load of emergency physicians during OHCA scenarios.

## 1. Introduction

Out-of-hospital cardiac arrests (OHCAs) have been associated with high incidence and mortality rates, making it one of the leading causes of death across Europe [1]. Aside from adequate cardiopulmonary resuscitation (CPR) and defibrillation, early identification and treatment of reversible causes are key to further improving the chances of survival following a cardiac arrest. As a central part of the Advanced Life Support (ALS) algorithm, these reversible causes, which include hypoxia, hypovolemia, hypo-/hyperkalemia and metabolic derangement, hypo-/hyperthermia, coronary or pulmonary thrombosis, tension pneumothorax, cardiac tamponade, and toxins [2]—commonly referred to as the “4 Hs and 4 Ts”—need to be ruled out.

Concerning the ALS algorithm, the European Resuscitation Council (ERC) guidelines consider the use of point-of-care ultrasound (POCUS) for the diagnosis of treatable causes, such as tension pneumothorax or cardiac tamponade. Furthermore, blood gas analysis can help determine abnormal blood glucose levels or the presence of hypo-/hyperkalemia. In cases of presumed intoxication, the toxic agent should be identified as early as possible [2,3]. Indeed, one study showed that patients with OHCA who underwent both POCUS and point-of-care blood testing received cause-specific prehospital treatment more frequently than did those who did not [4]. Furthermore, identification of the underlying cause of the OHCA was associated with a significantly increased probability of return of spontaneous circulation (ROSC) [5].

The ERC guidelines further recommend designating a team leader to manage the team and ensure adherence to the ALS algorithm, as well as detecting and treating potential reversible causes concurrently [3]. However, such scenarios often pose considerable challenges, particularly for the team leader, given the multitude of demanding tasks, resulting in a high cognitive load. This heightened cognitive load can further negatively affect the ability to process new information, which negatively affects CPR performance [6,7]. According to a recent study by Dewolf et al., the proposed “4 Hs and 4 Ts” were utilized in only four of five evaluations during ALS, whereas cause-specific investigations were performed in only 75% of resuscitations [5].

Over the past years, various checklists and electronic clinical decision support (eCDS) tools have been developed and tested for usability and reliability in helping medical professionals treat patients in critical situations [8,9,10,11]. Evidence has shown that the utilization of these devices could lower cognitive load during decision-making and positively impact guideline adherence [8,10]. Furthermore, improved adherence to ALS guidelines could increase the probability of achieving ROSC by treating reversible causes of the cardiac arrest [12]. Despite the established mnemonics for reversible causes of cardiac arrest, no standardized protocol has yet been established to guide their structured assessment during CPR [4,5]. Furthermore, the structured processing of reversible causes during CPR can be challenging due to the environment, task demand, human factors, or patient-related complexity. Additionally, according to Dewolf et al., the underlying causes of cardiac arrests are even less frequently identified in OHCAs than in in-hospital cardiac arrests [5]. In such cases, a structured checklist could enable a complete and efficient assessment of reversible causes.

The current study therefore aimed to assess the usability and applicability of a mobile checklist developed to aid in the detection of reversible causes of OHCA. A digital checklist might help reduce the cognitive workload of team leaders and ensure that all potentially reversible causes have been ruled out.

## 2. Materials and Methods

### 2.1. Study Design

This study employed a mixed-methods approach with two sequential phases. In phase I, a focus group was conducted to draft an interactive mobile checklist for reversible causes of OHCA. Furthermore, its applicability and utility were assessed through questionnaires based on the mHealth App Usability Questionnaire [13], which was used as a template but still required adaptation. After all responses were collected, the average score was calculated, with higher scores indicating better usability.

Our study population consisted of 20 emergency physicians from the Medical University of Vienna, Austria.

This study was performed in cooperation with the Department of Anesthesia, Intensive Care Medicine and Pain Medicine of the Medical University of Vienna, Austria, the Ludwig Boltzmann Institute Digital Health and Patient Safety, Vienna, Austria, and the Emergency Medical Service Vienna, Austria.

This study was approved by the local Ethics Committee of the Medical University of Vienna (1608/2022, approved on 16 September 2022, chaired by Martin Brunner). The data protection declaration was also approved by the intra-university data protection committee of the Medical University of Vienna. All participants provided informed consent prior to study participation.

### 2.2. Study Setting

Home to over 2 million people, the capital city of Austria, Vienna, has an annual treated OHCA incidence of approximately 77.1/100,000 population per year. The city’s ambulance service is run by emergency medical services (EMS) Vienna, which covers all emergency calls 24 h per day together with partner organizations. In case of life-threatening emergencies, emergency physician attendance is mandatory. Until 1 out of the 10 physician-staffed rapid response vehicles arrive, well-trained emergency medical technicians (EMTs) provide ALS on their own [14]. Besides first responders from both the police and fire departments, a senior EMT, known as the “field supervisor,” as well as a research vehicle staffed with an EMT and an emergency physician, are dispatched [15,16].

Each physician-staffed rapid response vehicle in Vienna city is equipped with a mobile ultrasound device and a mobile blood gas analyzer, which enables state-of-the-art treatment according to the guidelines by bringing point-of-care treatment to the scene.

#### 2.2.1. Phase I

All emergency physicians from the Medical University of Vienna, Department of Anesthesia, Intensive Care Medicine and Pain Medicine were invited to join a 2 h focus group. Nine emergency physicians from the Medical University of Vienna and one senior physician from the EMS Vienna participated in a moderated group discussion. Suggestions, requests, and concerns of the participants were collated anonymously. In the second step, the study team created an interactive checklist to rule out reversible causes of OHCA based on the focus group results. None of the participants received financial compensation.

#### 2.2.2. Phase II

Subsequently, 20 emergency physicians from the Medical University of Vienna, Department of Anesthesia, Intensive Care Medicine evaluated this interactive checklist. For this reason, each physician received the mobile version of the checklist on a tablet and familiarized its various features. To assess applicability and user friendliness, as well as potential for improvement, the participants completed the MAUQ-based questionnaire [13], which was validated for the usability of mHealth app. Given that not all subthemes were relevant for the developed checklist, the questionnaire was adapted. The entire questionnaire is provided in the Appendix B Supplements (Study questionnaire).

### 2.3. Study Population

All participants held an active diploma in prehospital emergency medicine from the Austrian Medical Chamber and were either a specialist or resident in anesthesiology and intensive care medicine with at least 36 months of professional experience.

### 2.4. Statistical Methods

Baseline characteristics were presented as median values with interquartile range for continuous variables and proportions for categorical variables. Results from the evaluation process were presented as mean values and total scores, which were visualized using Likert scales. Standard deviations (SD) and 95% confidence intervals (CI) were calculated to describe response variability and the precision of the mean values. Moreover, Likert responses were presented as absolute and relative frequencies.

Owing to the small sample size, subgroup analysis was performed for exploratory purposes only and should therefore be interpreted cautiously. Differences in response distributions between specialist and resident physicians were assessed using Fisher’s exact test and subsequently described descriptively.

All statistical analyses were conducted using the R software package (version 3.6.1, R Foundation for Statistical Computing, Vienna, Austria). Figures and tables were created using Microsoft Word^®^ and RStudio^®^ (version 2023.9.1.494, Posit Software, RStudio: Integrated Development Environment for R, Boston, MA, USA).

### 2.5. Data Collection

Pseudonymized data collection was employed using case numbers and stored password protected at the Department of Anesthesia, Intensive Care Medicine, and Pain Medicine, Medical University of Vienna, Austria.

## 3. Results

### 3.1. Phase I

#### 3.1.1. Focus Group

As part of the focus group, a moderated 2 h group discussion focusing on key points included in the checklist took place. In particular, the focus group addressed the aspects covered by the checklist and the appropriate level of detail, its integration with the existing mobile POCUS application, and the standard operating procedures of the EMS Vienna. All participants agreed to participate in the focus group. Thereafter, the study team took notes from the discussion and drafted the checklist based on the participants’ comments.

#### 3.1.2. Development Process

**Figure 1:**
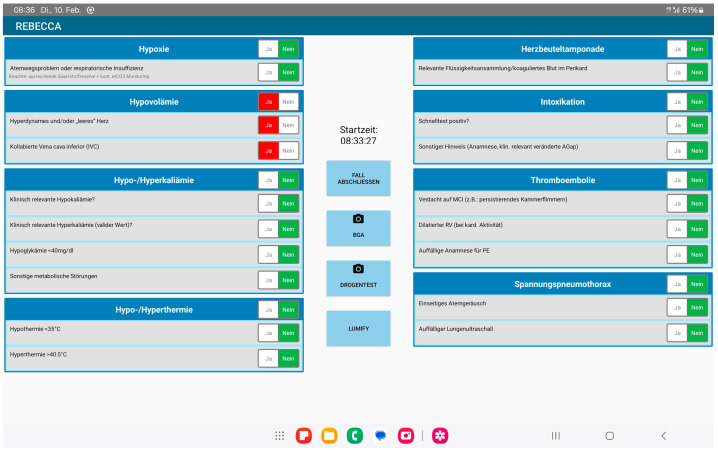
Overview of the user interface of the mobile checklist on the tablet. The screenshot shows the main screen of the reversible causes of out-of-hospital cardiac arrest checklist during patient evaluation, with hypovolemia an example of the reversible cause. After clicking the first button, the clock will start automatically. The checklist is completed once all boxes have been completed. An English translation of the checklist is available from the corresponding author upon request.

The study team drafted the digital checklist in German. As demonstrated in Figure 1, the checklist consists of a single page with the “4 Hs” listed on one side and the “4 Ts” listed on the other. An electronic clock, a button to end and save the case, and buttons to scan the blood gas and drug test results were placed between the lists. The last button links the POCUS app directly to the interactive checklist.

Each reversible cause is listed with the specific questions that need to be ticked off (“yes” or “no”) when ruling out each potential origin of cardiac arrest. Selecting “no” indicates that this particular characteristic could not be monitored, whereas selecting “yes” will highlight the button in red indicating the possible presence of this specific reversible cause.

#### 3.1.3. The “4 Hs”

To address hypoxia as the reversible cause, the first question determines whether any issue concerning the airway or respiratory insufficiency is present. A note reminds the user to monitor the oxygen reservoir and etCO_2_. To rule out hypovolemia, the user must evaluate (1) whether the heart appears hyperdynamic and/or “empty” (missing blood filling) and (2) whether the inferior vena cava has collapsed. The third box on the left addresses hypo-/hyperkalemia, hypoglycemia, and other metabolic abnormalities. The first two questions evaluate abnormal potassium levels, with caution regarding validity in hyperkalemia. The last box on the left evaluates hypo-/hyperthermia (<35.0 °C; >40.5 °C).

#### 3.1.4. The “4 Ts”

The box on the right top assesses the presence of cardiac tamponade, which would require POCUS. The second box focuses on the diagnosis of an intoxication through the drug testing and further information that could likely indicate intoxication (e.g., medical history and a significantly increased anion gap). Third, the probability of thromboembolism is evaluated by checking for signs of myocardial infarction (e.g., persistent ventricular fibrillation) and those suspected of having a pulmonary embolism (PE) (e.g., medical history, immobility, or echocardiographic right ventricular dilation). The box on the bottom right side recommends ruling out tension pneumothorax via auscultation of breath sounds, with the absence of ventilation on either side potentially indicating pneumothorax. In intubated patients, attention is focused on unintended single lung ventilation. Additionally, the checklist requires the emergency physician to observe for signs of pneumothorax when performing POCUS (e.g., bar code sign and absent pleural sliding).

### 3.2. Phase II

#### 3.2.1. Basic Characteristics

The basic characteristics of the participants are summarized in Table 1. Among the 20 participants (median age, 34.5 [32.8–38.0] years), 15 were male (75%). Moreover, 6 of the 20 emergency physicians were resident physicians (30%), with the majority of the participants being specialists in anesthesia and intensive care medicine. The median work experience as an emergency physician in the prehospital field was 2.0 (1.0–4.3) years. The interviewed emergency physicians worked between 1 and 6 shifts per month (median, 4.0 [3.4–6.0]) on the emergency physician unit.

**Figure 2:**
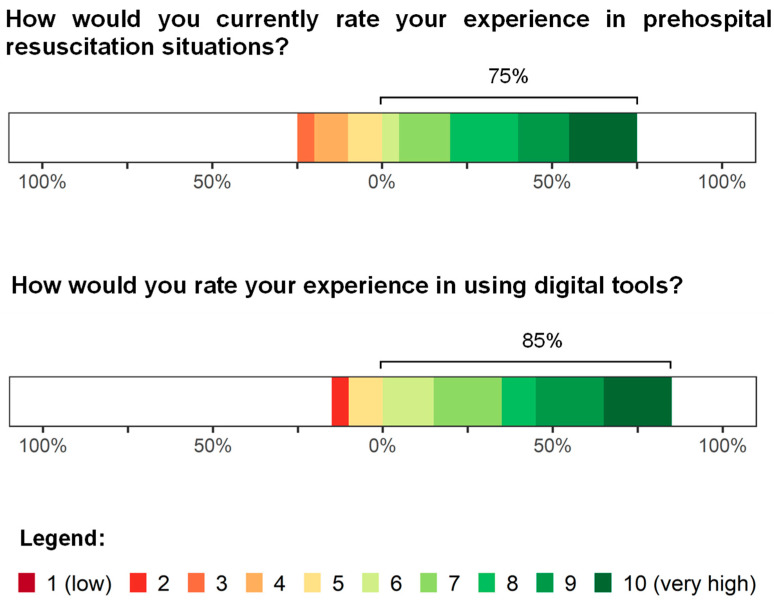
Assessment of the level of experience with out-of-hospital cardiac arrest resuscitation situations and confidence with digital tools. Responses were recorded using a 10-point Likert scale ranging from 1 (low; red) to 10 (very high; green).

In the first part of our questionnaire, the participants were asked to estimate their own levels of experience with digital tools and management of OHCAs. Accordingly, 75% of the participants provided a rating of 5 or higher regarding their current experience with out-of-hospital resuscitation situations, with an average of 7.35 out of 10 (Figure 2). When asked about their expertise in handling digital tools, 85% provided an experience rating situated in the upper half of the scale (7.5 out of 10). Table 2 presents the corresponding summary statistics (mean, SD, and 95% CIs), whereas Appendix A Table A1 lists the distribution of responses (n, %).

#### 3.2.2. Checklist Evaluation

The participants were then asked to evaluate the interactive checklist for usability, with most providing consistent ratings. The first few questions primarily focused on evaluating the usefulness of the checklist and whether the participants would use the checklist during OHCA resuscitations at all.

**Figure 3:**
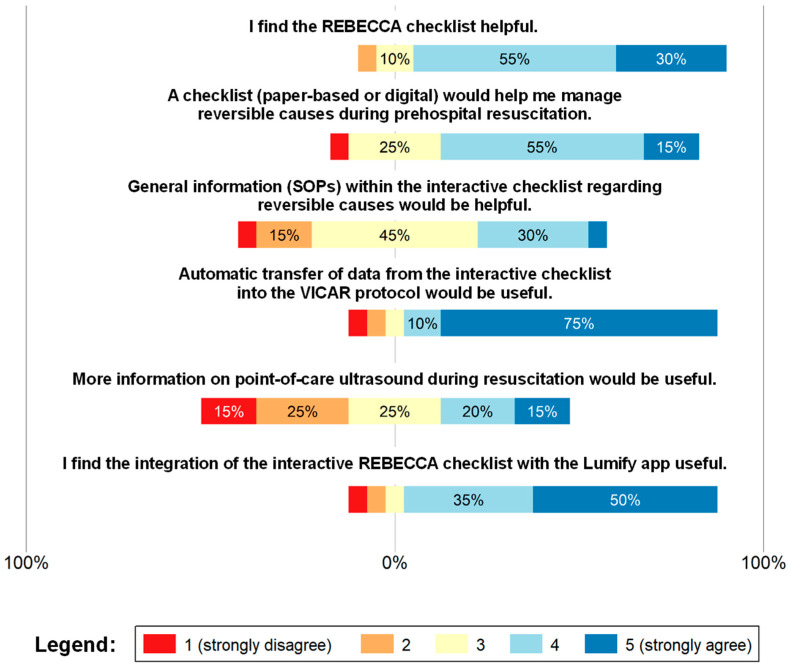
Assessment of the Usefulness of the digital checklist. Responses were recorded using a 5-point Likert scale ranging from 1 (strongly disagree; red) to 5 (strongly agree; blue). Intermediate values represent increasing levels of agreement, with 3 (yellow) indicating a neutral response. Abbreviations: SOPs, standard operation procedures; VICAR, Vienna Cardiac Arrest Registry. The Philips^®^ Lumify app 5.1.1 300016789831 25-12-14 was the point-of-care ultrasound application used by EMS Vienna at the time of evaluation.

As shown in Figure 3, 85% thought that the checklist was a helpful feature (4.1 of 5), with a majority of physicians believing that a checklist would be helpful in determining the reversible causes of OHCA during resuscitation management (3.75 of 5). However, no clear consensus was reached on whether standard operating procedures (SOPs) should be included in the checklist setup. The majority of participants (85%) stated that they would have appreciated automated data transfer between the checklist and the Vienna cardiac arrest registry protocol. Although 17 of the 20 participants stated that linking the POCUS app directly to the checklist would be a useful feature, responses concerning the need for additional information on POCUS during resuscitation were almost equally distributed (2.95 of 5). Table 3 details the corresponding summary statistics (mean, SD, and 95% confidence intervals), whereas Appendix A Table A2 presents the distribution of responses (n, %).

**Figure 4:**
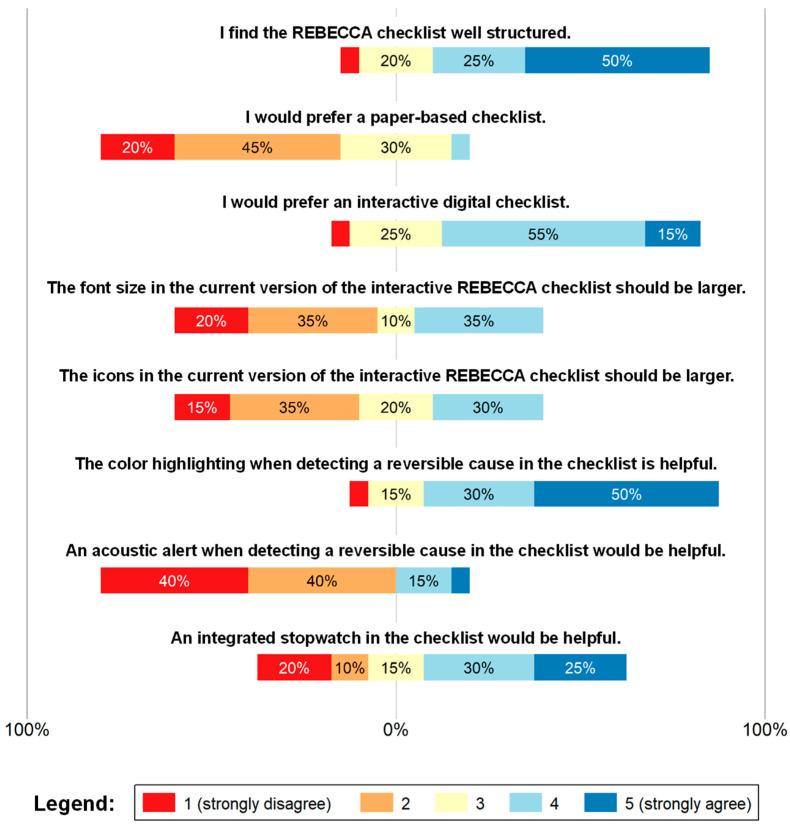
Assessment of the ease of use and satisfaction with the interface of the digital checklist. Responses were recorded using a 5-point Likert scale ranging from 1 (strongly disagree; red) to 5 (strongly agree; blue). Intermediate values represent increasing levels of agreement, with 3 (yellow) indicating a neutral response.

In the second part, ease of use and satisfaction with the checklist interface were evaluated (Figure 4). Accordingly, 50% of the emergency physicians stated that the checklist was well-structured, whereas only one participant did not agree at all (4.15 of 5). Concerning the type of checklist, participants clearly preferred an interactive and digital checklist over a paper-based checklist. Responses regarding both the font size and icons size were almost equal and did not follow any clear trend. The majority of the participants considered color coding (red and green) a helpful feature when a reversible cause was detected. A total of 80% stated that this implementation was meaningful (4.2 of 5), with only one participant not agreeing and three responding neutrally. When asked whether an acoustic signal would be helpful when assessing a reversible cause, completely opposite results were found (2.05 of 5). No clear results were obtained on whether an electronic clock should be included in the checklist setup. Table 4 lists the corresponding summary statistics, whereas Appendix A Table A3 details the distribution of responses (n, %).

**Figure 5:**
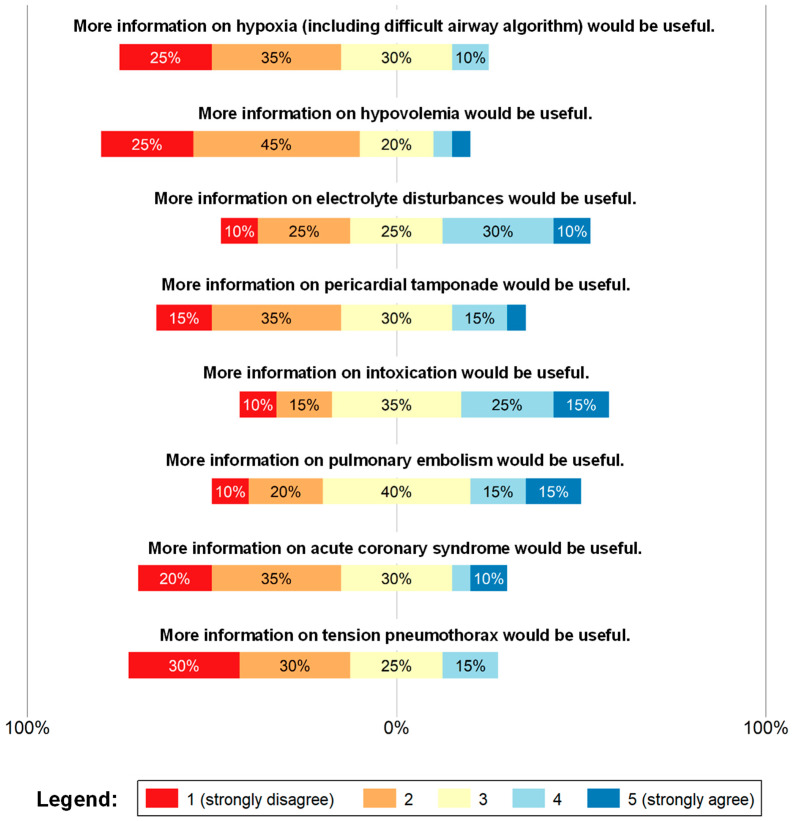
Assessment of the satisfaction with the information on the reversible causes within the checklist. Responses were recorded using a 5-point Likert scale ranging from 1 (strongly disagree; red) to 5 (strongly agree; blue). Intermediate values represent increasing levels of agreement, with 3 (yellow) indicating a neutral response.

The third part of the questionnaire focused on the satisfaction with the information provided on the reversible causes (Figure 5). A total of 60–70% stated that additional information on hypoxia, tension pneumothorax, and hypovolemia wouldn’t be necessary. While there was no need for further information on acute coronary syndrome (ACS) and cardiac tamponade, results for electrolyte disorders or intoxication remained unclear. On behalf of pulmonary embolism, 40% of the participants voted neutral (3.05 of 5). The corresponding summary statistics are shown in Table 5, whereas the distribution of responses (n, %) is listed in Appendix A, Table A4.

#### 3.2.3. Subgroup Analysis

A subgroup analysis was conducted by dividing participants into residents (n = 6) and specialists (n = 14). For most questions, no significant differences in response distributions could be identified. Nevertheless, differences could be observed for the following two questions, which have been presented in Table 6. However, given the small sample size, this subgroup analysis was solely exploratory.

## 4. Discussion

Emergency physicians provided positive feedback regarding the design and features of the interactive checklist, especially the linking of the checklist to the POCUS app and local cardiac arrest registry protocol. In particular, creating a link between the checklist and local cardiac arrest registry protocol allows for the transmission of patient data and information about the cardiac arrest directly from the scene to the receiving hospital, provided that an appropriate interface is established. Considering the high level of training among the participating physicians, the excellent self-assessment of their OHCA management expertise (7.35 out of 10) was unsurprising. Nevertheless, the majority of the participating physicians found the checklist to be helpful and carefully structured. The Vienna EMS is a resource-rich (POCUS, blood gas) and physician-based EMS. Especially in this setting, the participants found the linking of the checklist to the POCUS app and the local cardiac arrest registry protocol practical, given that it could simplify the working process and potentially save time during demanding CPR scenarios. However, the performance of nonphysician EMS might differ. Furthermore, they mostly agreed that the checklist was generally well designed and that certain features regarding the user interface (e.g., color coding, font size, and adding a clock to the main screen) met their needs. These findings provide essential information on lowering subjective cognitive load, which helps emergency physicians function efficiently in often hectic and unorganized situations on scene. This checklist could guide team leaders and potentially nonphysician EMS by helping them work through all relevant tasks during ALS in OHCA, potentially improving patient outcomes [6,8,10].

When asked about satisfaction concerning information about the “4 Hs and 4 Ts,” the responses were almost consistent. Although physicians stated that no further information on most of the reversible causes was needed, a small majority of the respondents wished for more details on electrolyte disorders and intoxication. One reason might be the fact that both electrolyte disorders and intoxications are relatively rare causes of OHCAs compared to ischemic/structural cardiac and respiratory etiologies [17,18,19]. Although opioids, alcohol, and tricyclic antidepressants have been most frequently associated with intoxications identified during cardiac arrest in Austria, almost half of the cases have been reported to involve mixed intoxications with several substances [20].

Regarding metabolic disorders, hyperkalemia has been considered the most relevant potentially reversible cause of OHCAs and does not exclusively occur in patients suffering from renal failure [3]. Team leaders may find it difficult to determine whether the electrolyte disorder is the underlying cause or a symptom of the cardiac arrest; however, in most cases, it will still be treated.

A small majority of physicians also expressed the need for additional information on PE within the checklist, which only lists right ventricular dilation and indicative medical history. PE has been identified as a frequent cause of cardiac arrest that is potentially being underestimated due to the lack of clear diagnostic criteria for OHCAs [21,22]. In patients with spontaneous circulation, dilation of the right ventricle serves as an important diagnostic indicator of PE, which could be the cause of cardiac arrest [3].

### Limitations

The single-center design of this study and the small sample size limit the generalizability and transferability of its findings, particularly regarding the structural and historical differences across EMS systems worldwide. Accordingly, acceptance and feasibility of the checklist among EMS providers outside Vienna may be difficult to predict. Although emergency physicians with different levels of experience were included in this study, the sample size still remains a limitation. Another limitation is that the focus group discussion was not audio-recorded; therefore, no transcription was possible. However, detailed notes on the findings of the moderation had been taken. These findings were then discussed among the study team and provided the basis for the creation of the checklist.

This feasibility and usability study did not perform cognitive load or time measurements and did not assess the performance of the REBECCA checklist in actual or simulated OHCAs or evaluated its outcomes.

## 5. Conclusions

Regardless of experience, the majority of emergency physicians included in this study appreciated the presented REBECCA checklist developed to determine the reversible causes of OHCA. Notably, questionnaire responses showed a small trend toward requesting more details on relatively rare reversible causes of OHCA, which may reflect their often versatile and different presentation, making diagnosis even more difficult. Further validation through randomized controlled trials will be needed to determine the potential benefits of the REBECCA checklist.

## Figures and Tables

**Table 1:** Basic characteristics.

Characteristics	Total, N = 20 ^1^
Sex	
Female	5/20 (25%)
Male	15/20 (75%)
Age (years)	34.5 (32.8–38.0)
Level of training	
Resident physician	6/20 (30%)
Specialist physician	14/20 (70%)
Experience as an emergency physician (years)	2.0 (1.0–4.3)
Shifts (per month)	4.0 (3.4–6.0)

^1^ n/N (%) or median (IQR).

**Table 2:** Summary statistics of responses regarding the level of experience with prehospital resuscitation situations and experience with digital tools.

Item	Mean	SD	95% CI
How would you currently rate your experience in prehospital resuscitation situations?	7.35	2.21	6.32–8.38
How would you rate your experience using digital tools?	7.50	2.12	6.51–8.49

Reported values were rounded off to two decimal places.

**Table 3:** Summary statistics regarding the usefulness of the digital checklist.

Item	Mean	SD	95% CI
I find the REBECCA checklist helpful.	4.1	0.79	3.73–4.47
A checklist (paper-based or digital) would help me manage reversible causes during prehospital resuscitation.	3.75	0.91	3.32–4.18
General information (SOPs) within the interactive checklist regarding reversible causes would be helpful.	3.15	0.93	2.71–3.59
Automatic transfer of data from the interactive checklist into the VICAR protocol would be useful.	4.45	1.15	3.91–4.99
More information on point-of-care ultrasound during resuscitation would be useful.	2.95	1.32	2.33–3.57
I find the integration of the interactive REBECCA checklist with the Lumify app useful.	4.2	1.11	3.68–4.72

Reported values were rounded off to two decimal places.

**Table 4:** Summary statistics regarding ease of use and satisfaction with the interface of the digital checklist.

Item	Mean	SD	95% CI
I find the REBECCA checklist well structured.	4.15	1.09	3.64–4.66
I would prefer a paper-based checklist.	2.2	0.83	1.81–2.59
I would prefer an interactive digital checklist.	3.75	0.91	3.32–4.18
The font size in the current version of the interactive REBECCA checklist should be larger.	2.6	1.19	2.04–3.16
The icons in the current version of the interactive REBECCA checklist should be larger.	2.65	1.09	2.14–3.16
The color highlighting when detecting a reversible cause in the checklist is helpful.	4.2	1.06	3.71–4.69
An acoustic alert when detecting a reversible cause in the checklist would be helpful.	2.05	1.23	1.47–2.63
An integrated stopwatch in the checklist would be helpful.	3.3	1.49	2.60–4.00

Reported values were rounded off to two decimal places.

**Table 5:** Summary statistics regarding the satisfaction with the information on the reversible causes within the checklist.

Item	Mean	SD	95% CI
More information on hypoxia (including difficult airway algorithm) would be useful.	2.25	0.97	1.80–2.70
More information on hypovolemia would be useful.	2.2	1.06	1.71–2.69
More information on electrolyte disturbances would be useful.	3.05	1.19	2.49–3.61
More information on pericardial tamponade would be useful.	2.6	1.10	2.09–3.11
More information on intoxication would be useful.	3.2	1.20	2.64–3.76
More information on pulmonary embolism would be useful.	3.05	1.19	2.49–3.61
More information on acute coronary syndrome would be useful.	2.5	1.19	1.94–3.06
More information on tension pneumothorax would be useful.	2.25	1.07	1.75–2.75

Reported values were rounded off to two decimal places.

**Table 6:** Differences in response distributions between resident (R) and specialist (S) physicians. The responses per group are presented per percentage.

Item	Training Level	1	2	3	4	5	*p* *
I would prefer a paper-based checklist.	R (n = 6)		100%				0.015
	S (n = 14)	28.6%	21.4%	42.9%	7.1%		
More information on hypoxia (including difficult airway algorithm) would be useful.	R (n = 6)	16.7%	50%		33.3%		0.045
	S (n = 14)	28.6%	28.6%	42.9%			

* *p* value for Fisher’s exact test.

## Data Availability

The data presented in this study are available on request from the corresponding author.

## Abbreviations

The following abbreviations are used in this manuscript:

ACS	Acute coronary syndrome
ALS	Advanced Life Support
CPR	Cardiopulmonary resuscitation
eCDS	Electronic clinical decision support
eCPR	Extracorporeal cardiopulmonary resuscitation
EMS	Emergency medical services
EMTs	Emergency medical technicians
ERC	European Resuscitation Counsil
MAUQ	mHealth App Usability Questionnaire
OHCA	Out-of-hospital cardiac arrest
PE	Pulmonary embolism
POCUS	Point-of-care ultrasound
REBECCA	Reversible causes of out-of-hospital cardiac arrest
ROSC	Return of spontaneous circulation
SOPs	Standard operating procedures
VICAR	Vienna Cardiac Arrest Registry

## Appendix A. Table A1, Table A2, Table A3 and Table A4

**Table A1:** Participant responses on the Likert scale items corresponding to the data shown in Figure 2: Assessment of the level of experience with out-of-hospital cardiac arrest resuscitation situations and confidence with digital tools. Results are presented as absolute and relative frequencies. Responses were recorded using a 10-point Likert scale ranging from 1 (low) to 10 (very high). Abbreviations: OHCA, out-of-hospital cardiac arrest.

Item	1	2	3	4	5	6	7	8	9	10
How would you currently rate your experience in prehospital resuscitation situations?	0	0	1 (5%)	2 (10%)	2 (10%)	1 (5%)	3 (15%)	4 (20%)	3 (15%)	4 (20%)
How would you rate your experience in using digital tools?	0	1 (5%)	0	0	2 (10%)	3 (15%)	4 (20%)	2 (10%)	4 (20%)	4 (20%)

**Table A2:** Participant responses on the Likert scale items corresponding to the data shown in Figure 3: Assessment of the Usefulness of the digital checklist. Results are presented as absolute and relative frequencies. Responses were recorded using a 5-point Likert scale ranging from 1 (strongly disagree) to 5 (strongly agree). Abbreviations: OHCA, out-of-hospital cardiac arrest; SOPs, standard operating procedures; VICAR, Vienna cardiac arrest registry; POCUS, point-of-care ultrasound.

Item	1	2	3	4	5
I find the REBECCA checklist helpful.	0	1 (5%)	2 (10%)	11 (55%)	6 (30%)
A checklist (paper-based or digital) would help me manage reversible causes during prehospital resuscitation.	1 (5%)	0	5 (25%)	11 (55%)	3 (15%)
General information (SOPs) within the interactive checklist regarding reversible causes would be helpful.	1 (5%)	3 (15%)	9 (45%)	6 (30%)	1 (5%)
Automatic transfer of data from the interactive checklist into the VICAR protocol would be useful.	1 (5%)	1 (5%)	1 (5%)	2 (10%)	15 (75%)
More information on point-of-care ultrasound during resuscitation would be useful.	3 (15%)	5 (25%)	5 (25%)	4 (20%)	3 (15%)
I find the integration of the interactive REBECCA checklist with the Lumify app useful.	1 (5%)	1 (5%)	1 (5%)	7 (35%)	10 (50%)

**Table A3:** Participant responses on the Likert scale items corresponding to the data shown in Figure 4: Assessment of ease of use and satisfaction with the interface of the digital checklist. Results are presented as absolute and relative frequencies. Responses were recorded using a five-point Likert scale ranging from 1 (strongly disagree) to 5 (strongly agree).

Item	1	2	3	4	5
I find the REBECCA checklist well structured.	1 (5%)	0	4 (20%)	5 (25%)	10 (50%)
I would prefer a paper-based checklist.	4 (20%)	9 (45%)	6 (30%)	1 (5%)	0
I would prefer an interactive digital checklist.	1 (5%)	0	5 (25%)	11 (55%)	3 (15%)
The font size in the current version of the interactive REBECCA checklist should be larger.	4 (20%)	7 (35%)	2 (10%)	7 (35%)	0
The icons in the current version of the interactive REBECCA checklist should be larger.	3 (15%)	7 (35%)	4 (20%)	6 (30%)	0
The color highlighting when detecting a reversible cause in the checklist is helpful.	1 (5%)	0	3 (15%)	6 (30%)	10 (50%)
An acoustic alert when detecting a reversible cause in the checklist would be helpful.	8 (40%)	8 (40%)	0	3 (15%)	1 (5%)
An integrated stopwatch in the checklist would be helpful.	4 (20%)	2 (10%)	3 (15%)	6 (30%)	5 (25%)

**Table A4:** Participant responses on the Likert scale items corresponding to the data shown in Figure 5: Assessment of satisfaction with the information on the reversible causes within the checklist. Results are presented as absolute and relative frequencies. Responses were recorded using a 5-point Likert scale ranging from 1 (strongly disagree) to 5 (strongly agree).

Item	1	2	3	4	5
More information on hypoxia (including difficult airway algorithm) would be useful.	5 (25%)	7 (35%)	6 (30%)	2 (10%)	0
More information on hypovolemia would be useful.	5 (25%)	9 (45%)	4 (20%)	1 (5%)	1 (5%)
More information on electrolyte disturbances would be useful.	2 (10%)	5 (25%)	5 (25%)	6 (30%)	2 (10%)
More information on pericardial tamponade would be useful.	3 (15%)	7 (35%)	6 (30%)	3 (15%)	1 (5%)
More information on intoxication would be useful.	2 (10%)	3 (15%)	7 (35%)	5 (25%)	3 (15%)
More information on pulmonary embolism would be useful.	2 (10%)	4 (20%)	8 (40%)	3 (15%)	3 (15%)
More information on acute coronary syndrome would be useful.	4 (20%)	7 (35%)	6 (30%)	1 (5%)	2 (10%)
More information on tension pneumothorax would be useful.	6 (30%)	6 (30%)	5 (25%)	3 (15%)	0

## Appendix B. Supplements (Study Questionnaire)

**Questionnaire ID: _________**
**Interactive Checklist (“REBECCA Checklist”) for the Structured Management of Reversible Causes During Prehospital Resuscitation**
Dear Participant,
Thank you very much for participating in this study.
This questionnaire is intended to evaluate the use of a structured, virtual checklist for addressing potential reversible causes during cardiopulmonary resuscitation in the prehospital setting among emergency physicians. In order to investigate possible associations with individual experience in using such tools, it is necessary to collect various characteristics, attributes, and experiences of participants. All data will be analyzed in a pseudonymized manner. Please complete the following questionnaire after familiarizing yourself with the interactive “REBECCA” checklist.
**Demographic Information**
1. How old are you?
  Age in years: ____
2. Which gender do you identify with?
  □ Male □ Female □ Diverse
3. What is your current level of training?
  Resident physician: Yes/No
  Specialist: Yes/No
4. How long have you been working as an emergency physician?
  Years: ____
5. Approximately how many emergency service shifts per month have you completed in the last six months?
  Number of shifts per month: ____
6. How would you currently rate your experience in prehospital resuscitation situations?
  Low to very high (1–10)
7. How would you rate your experience in using digital tools?
  Low to very high (1–10)
**Please indicate your level of agreement (1 = strongly disagree, 5 = strongly agree)**
A checklist (paper-based or digital) would help me manage reversible causes during prehospital resuscitation.

1	2	3	4	5

I would prefer a paper-based checklist.

1	2	3	4	5

I would prefer an interactive digital checklist.

1	2	3	4	5

I find the REBECCA checklist well structured.

1	2	3	4	5

I find the REBECCA checklist helpful.

1	2	3	4	5

The font size in the current version of the interactive REBECCA checklist should be larger.

1	2	3	4	5

The icons in the current version of the interactive REBECCA checklist should be larger.

1	2	3	4	5

I find the integration of the interactive REBECCA checklist with the Lumify app useful.

1	2	3	4	5

The color highlighting when detecting a reversible cause in the checklist is helpful.

1	2	3	4	5

An acoustic alert when detecting a reversible cause in the checklist would be helpful.

1	2	3	4	5

An integrated stopwatch in the checklist would be helpful.

1	2	3	4	5

General information (SOPs) within the interactive checklist regarding reversible causes would be helpful.

1	2	3	4	5

More information on hypoxia (including difficult airway algorithm) would be useful.

1	2	3	4	5

More information on tension pneumothorax would be useful.

1	2	3	4	5

More information on hypovolemia would be useful.

1	2	3	4	5

More information on pulmonary embolism would be useful.

1	2	3	4	5

More information on acute coronary syndrome would be useful.

1	2	3	4	5

More information on pericardial tamponade would be useful.

1	2	3	4	5

More information on electrolyte disturbances would be useful.

1	2	3	4	5

More information on intoxication would be useful.

1	2	3	4	5

More information on point-of-care ultrasound during resuscitation would be useful.

1	2	3	4	5

Automatic transfer of data from the interactive checklist into the VICAR protocol would be useful.

1	2	3	4	5

Questionnaire ‘REBECCA Checklist’—Version 1.1, 15 December 2023
